# A review on transition metal oxides in catalysis

**DOI:** 10.3389/fchem.2024.1374878

**Published:** 2024-05-17

**Authors:** Sanjubala Sahoo, Kaveendra Y. Wickramathilaka, Elsa Njeri, Dilshan Silva, Steven L. Suib

**Affiliations:** ^1^ Department of Materials Science and Engineering, Institute of Materials Science, University of Connecticut, Storrs, CT, United States; ^2^ Department of Chemistry, University of Connecticut, Storrs, CT, United States

**Keywords:** porous transition metal oxides, high temperature ceramic composites, coating, photocatalysis, electrocatalysis, density functional theory

## Abstract

Transition Metal Oxides (TMOs) have drawn significant attention due to their diverse range of properties and applications. The partially filled *d* orbitals of the transition metal ions, with highly electronegative oxygen atoms, give rise to unique electronic structures that lead to multiple applications due to their magnetic, optical, and structural properties. These properties have a direct influence on chemical reactions that enable tailoring materials for specific applications in catalysis, such as electrocatalysis and photocatalysis. While the potential of TMOs is promising, their development for enhanced functional properties poses numerous challenges. Among these challenges, identifying the appropriate synthesis processes and employing optimal characterization techniques are crucial. In this comprehensive review, an overview of recent trends and challenges in the synthesis and characterization of highly functional TMOs as well as ceramics will be covered with emphasis on catalytic applications. Mesoporous materials play a key role in augmenting their functionality for various applications and will be covered. Ab-initio modeling aspects for the design and development of novel TMO will be also discussed.

## 1 Introduction

Materials play a crucial role in the realms of chemistry, physics, and materials science, serving as the foundation for understanding the properties, behaviors, and applications of substances across these interdisciplinary fields. Transition metal oxides (TMOs) are one of the technologically important materials with wide ranges of applications such as in the chemical, energy storage and electronics industries where these are used as catalysts for the conversion of feedstocks to valuable chemicals and sensors, actuators (Nova, 2014). They are also used as electrode materials in electrochemical processes. In the electronics industry, TMOs are used as thin film conductors. Their use as catalysts is the most technologically advanced and economically important of all applications. Much progress has been made recently in the field of catalysis for the understanding of fundamental processes due to the development of advanced experimental and theoretical techniques which has made it possible to study the chemistry of the interface between the transition metal oxide and the fluid and gaseous phase.

The surface properties of TMOs, including acidity, basicity, and redox behavior, can be finely tuned through compositional modifications and surface functionalization (Cox, 2010). These tunable surface properties play an important role in modulation of catalytic activity, selectivity, and stability. For instance, the Lewis acid-base properties of TMOs facilitate the adsorption and activation of reactant molecules, while redox-active sites enable facile electron transfer processes during catalytic reactions. TMOs as supports have several advantages specifically for catalytic applications. Their thermal stability, chemical robustness, and resistance to sintering make them ideal candidates for high-temperature catalytic processes (Xu et al., 2020). TMO supports can provide redox-active sites that participate in catalytic cycles, promote oxygen activation, or stabilize reactive intermediates, thereby influencing reaction pathways and product distributions. By tailoring the surface chemistry of TMO supports, it is possible to optimize the interactions between the support and catalytic species thereby enhancing the overall performance of heterogeneous catalysts.

Recent developments in surface science techniques have provided more insight about the surface structures, chemical compositions, and electronic properties of the surfaces (Cox, 2010). Surface characterization probes include Low Energy Electron Diffraction (LEED) and Electron Energy Loss Spectroscopy (EELS) for investigating geometry and structure of surfaces, X-ray fluorescence (XRF) and X-ray photoelectron spectroscopy (XPS) for elemental compositions, chemical states, binding energies, and the electronic structures of surfaces. TMOs are projected as ideal candidates for single atom catalysis where the optimal efficiency could be gained by tuning catalyst support interactions along with quantum confinement effects of precious metal particles (Wang et al., 2018; Beniya and Higashi, 2019; Elsevier, 2019). The structure, thermal stability and wide range of surface functionalities provide highly stable supports for anchoring and stabilizing isolated metal atoms and fine dispersion. Moreover, the tunable electronic structures of TMOs lead to the precise control over the coordination environment and electronic properties of single metal atoms, facilitating the high catalytic activity and enhanced selectivity. A number of SACs have been synthesized using TMOs as supports for CO oxidation and methane conversion reactions (Qiao et al., 2011; Guo et al., 2014; Sahoo et al., 2016; Zuo et al., 2018). Among these, FeO_x_, Fe_2_O_3_, CeO_x_, TiO_2_ and zeolites supports together with Pt/Pd/Rh precious metal atoms are important (Bayram et al., 2015; Tang et al., 2019; Chen et al., 2022). TMOs are also used as precursors for catalysts besides being used as catalysts and support materials. For example, cobalt-molybdenum sulfide catalysts are used in hydrodesulfurization reactions where the catalyst is prepared by sulfiding cobalt-molybdenum oxide. Another example is chromium-based catalysts for ethylene polymerization. The catalyst can be made from supported chromium oxide as a precursor. Additionally, noble metal catalysts are prepared by reduction of the corresponding oxides, where the structures, morphologies, and properties of the TMO precursors have an influence on the properties of the final catalysts.

This review covers a combined experimental and theoretical overview of TMOs with emphasis on several synthetic processes, characterization methods, theoretical modeling approaches, and potential applications in photocatalysis, electrocatalysis and a variety of TMO catalyzed chemical reactions other than those related to photo and electrocatalysis. TMOs exhibit numerous structures, spanning from simple oxides to intricate mixed-metal oxides and nanostructured materials. Such structural diversity enables the precise control of surface morphology, porosity, and surface area, which have significant influence on catalytic performance. In this review, the primary focus will be on mesoporous TMOs due to their several advantages such as large surface areas, controlled pore sizes and structures, facile adsorption, and separation (Suib, 2017).

## 2 Synthesis and characterization of TMOs

Porous metal oxide materials are grouped into three classes based on pore size: microporous (less than 2 nm pore diameter), mesoporous (2–50 nm pore diameter), and macroporous with a pore diameter greater than 50 nm (Adegoke and Maxakato, 2022). Excellent reviews on porous TMOs and their applications have been published (Suib, 2017; Wang et al., 2017; Yang et al., 2017; Okonye et al., 2021). The seminal work on mesoporous materials by Mobil Corporation (Kresge et al., 1992) pioneered extensive research in the synthesis and utilization of porous TMOs. Nano-size mixed valent mesoporous oxides have received much attention in catalysis, electrochemistry, and adsorption due to their high surface areas and improved mass transfer within pores (Wang et al., 2017; Achola et al., 2022). Our lab has done considerable research on synthesizing mesoporous TMOs in recent years (Dang et al., 2021; Achola et al., 2022; Zhao et al., 2022a; Tabassum et al., 2022).

The synthesis methods of porous TMOs influence their crystallinity and porosity and thus their surface and bulk characteristics (Lermusiaux et al., 2022). Crystalline porous TMOs have different physicochemical properties compared to their amorphous counterparts. The presence of multiple crystalline phases in some oxides leads to distinct properties (Lermusiaux et al., 2022). In a study on the toxicity of TiO_2_ nanoparticles, Uboldi et al. (2016) confirmed that the rutile phase was slightly more toxic to cells than the anatase phase. The exposure of high energy crystal facets in TMOs results in improved gas sensing, photocatalytic activity, and selectivity in reactions (Zhou and Li, 2012). Crystal facet engineering can be accomplished during synthesis by selectively controlling the nucleation rate during crystal growth and utilizing capping agents (Liu et al., 2011; Zhou and Li, 2012). An example is the facet-controlled synthesis of h-WO_3_ for ppb level acetone detection via a fluorinated hydrothermal method. The highly active (002) facets and high density of oxygen vacancies resulted in superior sensing properties of acetone for the h-WO_3_ material mentioned above (Wang et al., 2019). Amorphous oxides present unique catalytic, optical, and electrochemical activity due to structural disorder and defects in these complex systems (Zhou and Fan, 2021). Nonetheless, a limitation in characterization techniques makes studying the structures of amorphous porous TMOs challenging.

While designing the porous materials, depending on the type of synthesis (top-down or bottom-up approach) and desired product, it is critical to have proper control over factors such as pH, temperature, solvents, precursors, type of surfactant, solubility in the reaction matrix, reaction time, scalability and so forth (Suib, 2017; Drummer et al., 2021). The environmental impact of these synthetic processes should also be evaluated since clean systems are essential to design intrinsically less hazardous systems that take advantage of greener technologies (Bandeira et al., 2020; Drummer et al., 2021; Zhao et al., 2022b). Reactions on the surface of TMOs are affected by their pore sizes. Thus, pore size control should be a key consideration for countless synthetic methods. Recent studies on hierarchically porous materials have shown that having multimodal pore size distribution (micropores, mesopores, and macropores) in TMOs improves catalytic and photocatalytic activity (Yang et al., 2017). Macroporous systems can enable better mass transportation of reactants to the active sites of porous TMOs in cases where mesoporous materials are limited by a smaller pore size (Li et al., 2015; Yang et al., 2017; Cai et al., 2021). Incorporating the strengths of different pore sizes into one material is a solution for improved mass transfer. Below are some recent examples of synthetic methods used to make porous TMOs.

**FIGURE 1:**
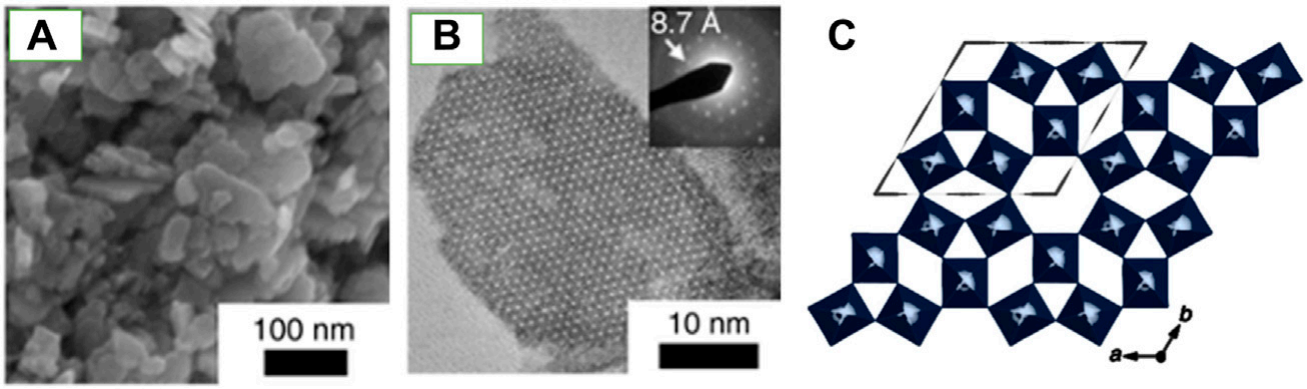
Structure and nanostructure of the h’-WO_3_ framework. **(A)** SEM and **(B)** HRTEM images showing nanosized platelets. **(C)** The tungsten octahedra along the c axis. Figure adapted from Ref. Lin et al. (2021).

Octahedral molecular sieves (OMS) are microporous oxide materials preferred for their mixed valent states and tunnel structures, facilitating superior redox chemistry. Incorporating several types of cations into transition metal OMS results in properties such as improved thermal stability and conductivity. The stability of OMS tunnel structures is dependent on water molecules or cations (Suib, 2008; Suib et al., 2020). These cations or water cannot be removed without collapsing the tunnels. A novel photosensitive h′WO_3_ OMS was synthesized by Besnardiere et al. (2019) via a “chimie douche” (soft chemistry) route. The synthesis initially yielded a mixed valent (W^V^, W^VI^) hexagonal hydrogen bronze h′-H_0.07_WO_3_, which converted to h′WO_3_ OMS with framework retention. In this case, the cations could be extracted reversibly via gentle annealing without collapsing the tunnels of the h′WO_3._ Characterization using powder X-Ray Diffraction (XRD), Scanning Electron Microscope (SEM), and high-resolution Scanning Transmission Electron Microscope (STEM) were utilized to identify the structure, crystal phase, and morphology of these OMS catalysts. Crystal defects were observed via STEM. The thermal stability of the materials was studied via thermogravimetric analysis (TGA) suggesting that the synthesized h′WO_3_ OMS have better thermal stability compared to h′WO_3_ previously reported in the literature. UV/Visible spectroscopy was used to analyze the optical properties of the OMS. Figures 1A–C illustrates the structure of the synthesized h′-WO_3_ framework (Besnardiere et al., 2019).

**FIGURE 2:**
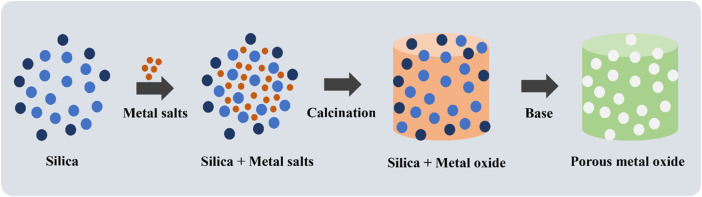
Mechanochemical nano casting route for the synthesis of porous metal oxide.

Mesoporous TMOs of Fe, Cu, Co and Zr were synthesized using a simple and fast solvent free mechanochemical nanocasting procedure (Xiao et al., 2018). The metal oxide precursors and silica template were ball milled in a stainless steel reactor. After calcination the powder was stirred in 2.5 M NaOH, at room temperature to remove the silica template to obtain a porous oxide. The surface areas and pore size distributions were investigated using N_2_ sorption studies and calculated via BET/BJH models, with reported surface areas as high as 293 m^2^/g. Surface morphology was studied using SEM, and the crystal structures were analyzed using powder XRD. The surface chemical state of ZrO_2_ analyzed through XPS confirmed the presence of oxygen vacancies and the primary oxidation state of Zr in the metal oxide (Zr^4+^). The materials were calcined at different temperatures and retained their porosity and high surface area at high calcination temperatures. In most cases, pores collapse under exposure to high calcination temperatures (Thalgaspitiya et al., 2020). Mechanical nanocasting can be a great alternative to traditional wet nanocasting syntheses, which are plagued with prolonged reaction times (hours) and restricted solubility of metal oxide precursors in solvents (Xiao et al., 2018). A comprehensive review of mechanical nanocasting discusses reaction mechanisms, choice of parameters and comparison to other synthesis methods (Tsuzuki, 2021). Figure 2 represents a schematic of the synthetic procedure (Xiao et al., 2018). By using co-solvents in a wet synthesis procedure, one can easily tune the pore sizes and crystal phases of porous TMOs. An example is the synthesis of Zr doped TiO_2_ by a modified inverse micelle route (Owalude et al., 2023). Both 1-butanol and hydrogen peroxide were used as solvents to synthesize a mixed phase (85% rutile and 15% anatase) TiO_2_ with mesopores and a small amount of macropores. The pore size distribution was determined by N_2_ sorption studies. Powder XRD studies confirmed that the anatase phase proportion increased to 36.8% upon Zr doping. The anatase/rutile phase % was determined from the XRD data. The crystalline phase was also confirmed using Raman spectroscopy. The catalysts were calcined at 450°C, a preferably low temperature for rutile formation. Optical studies were conducted using UV/Vis and photoluminescence spectroscopy (Owalude et al., 2023). Due to the poor solubility of precursors in a previously developed inverse micelle route (Poyraz et al., 2013), Thalgaspitiya et al. developed a metal dissolution synthetic procedure that involves dissolving metals in hydrogen peroxide to form peroxo complexes, followed by reacting the complexes with nitric acid and Pluronic P-123 surfactant in a one-pot synthesis. This approach led to the synthesis of multiple mixed valent mesoporous TMOs of Ti, Mn, Fe, Ni, Cu, and Zn, focusing on W and Mo oxides (Thalgaspitiya et al., 2020). A follow-up of this soft templating synthesis was done by using dual surfactants or solvent systems to form hierarchically porous TMOs (Yang et al., 2017). Photo-assisted synthetic methods such as photo-assisted sol gel syntheses or biodegradable templates were further explored to create more efficient and cleaner processes (Liu et al., 2003; Drummer et al., 2021). Some synthesis methods are sensitive to contamination from foreign particles, which could lead to undesirable materials. These syntheses could be done in a clean room to solve this issue. These reactions are typically done on an industrial scale but can also be applied to laboratory scale synthesis.

Some of the common challenges faced by synthetic chemists in metal oxide synthesis are precise control of particle size, scalability and reproducibly. Additionally, retention of framework structures while doping and phase identification via characterization play a major role in their performance as catalysts. To thoroughly investigate these features, *in-situ* techniques such as XRD and TEM are used. Phase changes along with varying temperature profiles have been studied. These methods are advantageous for correlating structure function relationships and studying mechanisms. The challenge of scalability and reproducibility is addressed by Besenhard et al. (2023) who used a high temperature flow reactor with varying temperature profiles to investigate the self-seeded growth mechanism of particle sizes <20 nm with temperature T < 200°C. They tried to keep the residence time to a minimum, which is a challenge to be performed under batch synthetic conditions. The goal of the synthesis is to use these materials as Magnetic Resonance Imaging (MRI) contrast agents or similar applications requiring high surface to volume ratios of iron oxide particles.

## 3 TMOs as photocatalysts

Photocatalytic water splitting reactions are widely researched due to their potential for providing clean and sustainable hydrogen. The process involves splitting water into its constituent elements (H_2_ and O_2_) in the presence of light and a photocatalyst, typically a semiconductor. To achieve maximum water splitting efficiency, the semiconductor valence band should be more positive than the water oxidation potential (O_2_/H_2_O, 1.23 eV), while the conduction band should be more negative than the hydrogen evolution potential (H^+^/H_2_, 0 eV). In addition, the photocatalyst should have an ideal band gap of >1.23 eV to facilitate efficient photon absorption (Lin et al., 2021; Bie et al., 2022). Nanoparticles are preferred due to their high surface area and faster diffusion (which occurs in the pico to nanosecond range) of photogenerated charges from the bulk to the surface of photocatalysts (Takanabe, 2017). Oxygen vacancies (Ov) enhance charge carrier separation, thus improving overall water splitting efficiency (Bie et al., 2022). Wei et al. synthesized tungsten oxides and Pt tungsten oxides with oxygen vacancies (O_v_-WO_3_ and O_v_-WO_3_-Pt, respectively) for photocatalytic oxygen evolution.

**FIGURE 3:**
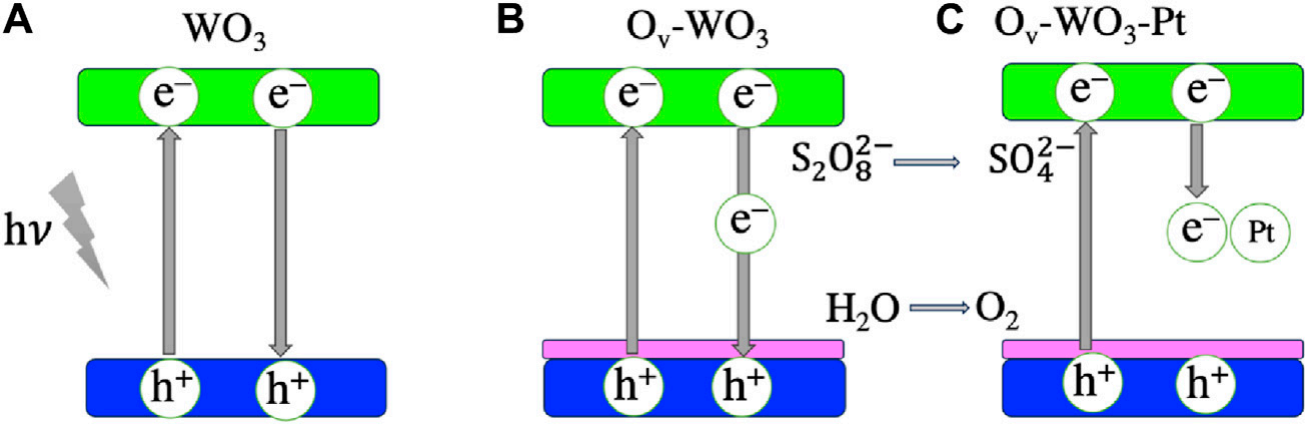
Mechanism of photoinduced electron transfer and charge recombination routes of **(A)** WO_3_, **(B)** Ov-WO_3_, and **(C)** Ov-WO_3_-Pt. The blue, green and magenta colors indicate the valence band, conduction band and surface oxygen-vacancy states, respectively. The figure is adapted from Ref. Zhang et al. (2018).

These samples were compared to pure WO_3._ XPS analysis indicated the presence of Ov due to the presence of W^5+^ in the W 4f core level spectra. An intense signal at g = 2.002 was observed for Ov-WO_3_ via Electron Paramagnetic Resonance (EPR) studies, confirming the XPS results. The charge carrier separation was investigated using femtosecond transient absorption spectroscopy and photoluminescence measurements. Photocatalytic oxygen evolution levels reached 683 µmolh^−1^ g^−1^, which is 4.3 times higher than pure WO_3._ The Pt cocatalyst further improved the photocatalytic performance of the O_v_-WO_3_ material. Figures 3A–C illustrates the mechanism of photoinduced electron transfer and charge recombination routes of WO_3_, Ov-WO_3_, and Ov-WO_3_-Pt (Wei et al., 2021).

Solar fuels such as methanol and methane can be produced through photocatalytic CO_2_ reduction. Forming composite materials through heterojunction engineering can drastically improve the reduction process (Li et al., 2021). An example is the enhanced redox and photon absorption efficiency achieved via Z-scheme heterojunctions. A TiO_2_/CdS direct Z-scheme thin film composite was successfully utilized for photocatalytic CO_2_ reduction and compared to TiO_2_ and CdS. Higher performance for methane production was observed for TiO_2_/CdS. Lesser amounts of methanol and formaldehyde were also detected using *in situ* Fourier Transform Infrared (FTIR) Spectroscopy. Electron transfer via a Z-scheme mechanism was confirmed using *in situ* irradiated XPS by observing the shifts in binding energy on the Ti 2p and Cd 3d spectra. ROS detection methods further confirmed the formation of a direct Z-scheme (Low J. et al., 2019).

An et al. (2022) report a direct photo oxidation approach to convert methane to methanol by using mono-iron hydroxyl sites. These iron hydroxyl sites are immobilized in a porous metal organic framework which can act as sites to promote C-H bond activation. Fe is incorporated into the framework by post synthetic metalation using FeCl_3_.6H_2_O precursor and the loadings determined by ICP-OES to be 2.6 wt%.

## 4 TMOs as electrocatalysts

Electrocatalysts are key components in energy storage and conversion devices such as fuel cells, where they facilitate the conversion of chemical energy into electrical energy through the oxidation of fuel such as hydrogen or methanol. In the electrolysis processes, electrocatalysts lead to the production of hydrogen or other valuable chemicals via water splitting. TMO-based electrocatalysis are of immense interest due to their diverse chemical compositions, tunable electronic structures, and catalytic activities. TMOs exhibit unique electrochemical properties that make them suitable for various applications, including oxygen reduction reactions (ORR) in fuel cells, oxygen evolution reactions (OER) in water-splitting devices, and carbon dioxide reduction reaction for sustainable fuel production.

The steam reforming of hydrocarbons still accounts for 50% of global hydrogen production, leading to global warming due to CO_2_ emissions. Electrochemical seawater splitting could be an abundant source of hydrogen, which is used as a fuel and chemical feedstock in various synthetic processes. The reaction involves hydrogen and oxygen evolution from the cathode and anode, respectively. However, the presence of chlorine ions and other impurities in seawater leads to electrode corrosion. A high overpotential is required for the OER thus limiting the efficiency of this process (Xiao et al., 2022). RuO_2_/NiO_2_ composite nanosheets were fabricated on nickel foam (RuO_2_-NiO NSs/NF) for seawater splitting to produce hydrogen through hydrogen evolution reaction (HER) under acidic and alkaline conditions. RuO_2_-NiO NSs/NF calcined at 300°C showed the best HER activity, higher than commercial Pt. The RuO_2_ was amorphous up to 300°C. During HER, the RuO_2_ formed an Ru/RuO_2_ interface, contributing to the performance of the catalysts. Dang et al. also hypothesized that the improved water adsorption and dissociation capabilities accelerated the Volmer step, subsequently leading to the production of H_2_. The stability of the catalysts was assessed through cycling experiments, resulting in excellent cycling durability compared to commercial Pt/C and Pt electrodes (Dang et al., 2021).

Fe containing oxides have been a primary candidate for electrochemical applications recently. Due to their low cost, non-toxicity and long-term stability, Iron containing compounds are preferred over expensive benchmark materials consisting of Platinum, Ruthenium, and Iridium. Wang et al. have reported the synthesis of partially amorphous, CoFeO_x_ by a facile ion exchange followed by an etching method (Wang et al., 2023a). Using a ZIF-67 framework as a structural template this method is developed and finally decorated with SO_4_^2−^ ions to enhance adsorption of reactants to the catalyst surface. The optimized nanosheets thus formed have proven to be effective OER catalyst with a greater stability (62 h) and an overpotential of 268 mV at 10 mA cm^−2^. On a similar note, NiFe_2_O_4_ synthesized using microwave assisted hydrothermal synthesis by Suib (Achola et al., 2022) has shown low overpotential of 278 mV at 10 mA cm^−2^ as an OER catalyst.

Among the TMOs, layered perovskites from the ABX_3_ family are reported to exhibit enhanced OER activities (Xu et al., 2020; Tang et al., 2022; Abdelghafar et al., 2024). Specifically, the layered Ruddleson-Popper perovskites such as Ba_0.5_Sr_0.5_Co_0.8_Fe_0.2_O_3–δ_ and LaSr_2.7_Co_1.5_Fe_1.5_O_10_ have demonstrated high OER activity with an overall high water splitting current density value 2.01 A cm^−2^ at 2 V at high temperature. The high OER activity is attributed due to the enhanced participation of lattice oxygen at high temperature.

Honeycomb like open edge reduced graphene oxide nanosheets (HOrGONSs) and TMO hybrids can be used as supercapacitors. NiO and Co_3_O_4_ are promising due to good electrochemical capacitance behavior, low cost, and ease of accessibility (Low W. H. et al., 2019). Kumar et al. synthesized a hybrid reduced graphene oxide/TMO system via a microwave method (HOrGO/TMO) for studies as electrode materials for supercapacitors. The TMO in this case was NiO/Co_3_O_4_. Reduced graphene oxide has exceptional electrical and thermal conductivity and a high specific surface area. Hybrid systems are expected to have superior conductivity and electrochemical capacitance behavior. The hybrid nanoparticles exhibited high electrochemical performance based on the electrochemical results. These data were influenced by the high surface area of the nanoparticles as well as the interaction between HOrGONSs and NiO/Co_3_O_4_ (Kumar et al., 2020).

Mesoporous Iron oxide synthesized using waste wood powder as a template has been successful in providing a high efficiency catalyst for the slurry phase hydrocracking of heavy oil. Ping et al. (2023) presents that by varying the NaOH content during synthesis both a-Fe_2_O_3_ and v-Fe_2_O_3_ can be obtained. Furthermore, the crystal size of the iron oxide reduces with increasing NaOH amounts. Larger surface areas close to 134 m^2^/g are recorded which provide more exposure of Fe active sites. These have proven to have improved hydrogenation activity with a conversion close to 80%. The mesoporous nature can enhance the diffusion of molecules in vacuum, allowing them to come into contact with the active sites. This promotes the conversion process, leading to a higher yield of gasoline and diesel distillates.

## 5 TMOs as general catalysts

Besides their application as photocatalysts and electrocatalysts, TMOs play a crucial role for catalyzing many other chemical reactions such as oxidations, selective oxidations, selective reductions, oxidative and non-oxidative dehydrogenations, water-gas shift reaction (WGS), CO_2_ hydrogenation, and many others. For example, FeO/Pt(111) and Cu_2_O/Ag(111) catalysts have demonstrated high reactivity for gas-phase aerobic oxidation of benzyl alcohol and other primary alcohols where the metal-oxide interface is identified as the active site (Zhao et al., 2017). Mixed valent manganese oxides have superior redox chemistry. They form layered, spinel, perovskite, bixbyte, and other structures with oxidation states ranging from 2^+^ to 4^+^ (Risch et al., 2017). MnO_2_@FeOOH catalysts were prepared for oxidation of indoor air containing formaldehyde by Wang C. et al. (2022) Reactive oxygen species (ROS) formed on the catalyst surfaces were responsible for formaldehyde oxidation. *In situ* and *ex situ* aqueous and gaseous ROS quenching experiments confirmed the crucial role of the superoxide anion in this oxidation. An ultrathin coating of MnO_2_ on FeOOH decreased electron transfer resistance while increasing the amount of oxygen vacancies on the surface of these materials. These oxygen vacancies facilitated the absorption of molecular oxygen, which was subsequently activated to generate ROS (Wang C. et al., 2022). Lithium promoted mesoporous manganese oxides were utilized for the mild partial oxidation of allyl ethers. Dutta et al. hypothesized that the reaction proceeded via a radical mediated route. Introducing lithium increased the surface activity, which improved catalytic activity. Excellent conversion (95%) to allyl acrylate and a selectivity of >99% were achieved for this reaction (Dutta et al., 2019). Al_2_O_3_ supported vanadia (VO_x_) and MoO_3_-Fe_2_O_3_ catalysts are reported to be highly active for the ODH of alkanes such as ethane and propane (Solsona et al., 2001; Kondratenko et al., 2005). Furthermore, the MoO_3_-Fe_2_O_3_ and ZnO_x_ catalysts are also reported to be highly active for the ODH of propane (Zhao et al., 2021; Wang et al., 2023b). IrO_2_-based catalysts have been highly effective for ethane oxidative dehydrogenation (Ping et al., 2023). In a recent study, the CeO_2-*x*_/CoO_1-*x*_/Co dual-interfaces are structurally active for catalyzing the WGS reaction, where the kinetic evidence and *in-situ* characterization results revealed that CeO_2-*x*_ modulates the oxidized state of Co species and consequently generates the dual active CeO_2-*x*_/CoO_1-*x*_/Co interface during the WGS reaction. The CeO_2-*x*_/CoO_1-*x*_ interface alleviates the CO poisoning effect, and the CoO_1-*x*_/Co interface promotes H_2_ formation (Fu et al., 2023). A combined Ni-NiO_*x*_-Y_2_O_3_ is reported to show high WGS activity when compared to pure Ni catalysts where the presence of Y_2_O_3_ tremendously improved the catalytic activity and stability, enabling efficient WGS reactivity at a medium temperature range (Xu et al., 2022). Many of the processes require high selectivity for specific products and oxidation of the reactant molecules. The multiple oxidation states of the oxide materials control the selectivity in catalytic oxidations using oxides. Some TMOs can also catalyze selective hydrogenation. For example, copper-zinc oxide-based catalysts are used as the primary catalyst rather than copper metal for the conversion of CO or CO_2_ via hydrogenation for methanol production (Martin and Pérez-Ramírez, 2013; Kattel et al., 2017a). Silica accelerates the selective hydrogenation of CO_2_ to methanol on cobalt catalysts (Wang et al., 2020).

Molybdenum based oxide materials are highly preferred in electrochemical applications due to their tunable crystal structures, variability in Mo oxidation states, and the ability to modify the composition by introducing dopants. Defect engineering also involves morphology and oxygen vacancies. Synthesizing a highly mesoporous MoO_x_ catalyst via an inverse micelle molybdenum peroxo cluster formation method has been reported (Shubhashish et al., 2022) where a high surface area of 157 m^2^/g is found. These systems have shown high catalytic activity towards electrophilic aromatic substitution of benzyl alcohol with a higher conversion and selectivity towards methyl diphenylmethane. Ammonia chemisorption has shown that these materials are highly acidic in nature which could be attributed to the higher electrophilicity of the material. The diffraction obtained from X-ray powder diffraction suggests that the material is orthorhombic phase MoO_3_.

**FIGURE 4:**
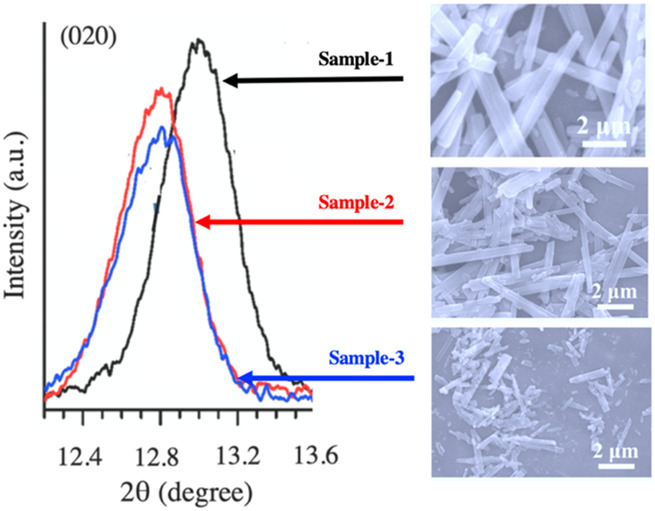
XRD patterns (left panel) and SEM images (right panel) of synthesized α-MoO_3_ with multiple etching durations. Sample-1 is the pristine MoO_3_ (no etching), Sample-2: etching for 10 min, Sample-3: etching for 20 min. The XRD patterns are plotted around (020) diffraction peak (Zhang et al., 2018). Figure adapted from Ref. Solsona et al. (2001).

α-MoO_3_ has been used as a cathode material in lithium-ion batteries due to its high specific capacity (<1.6 Li+ per one transition metal) where cycling causes irreversible phase changes in the material, resulting in lower performance. However, a synthetic route via controllable plasma etching of α-MoO_3_ has been performed by Zhang et al. (2018), which generates oxygen vacancies in the structure. The SEM image and XRD patterns of the synthesized pristine sample with varying etching times are demonstrated in Figure 4. Oxygen vacancies play a vital role in enhancing the electronic properties of these materials electronically through the band gap reduction which in turn affects the electron transfer resistance and effective Li^+^ diffusion. An incipient wet impregnation method has been used to synthesize crystalline MoO_3_ catalysts on Al_2_O_3_ supports by Yao et al. (2020) This material has proven to be effective in conversion of ethane into ethylene in the presence of oxygen as well as other mild oxidants. Ethylene selectivity is reported to be 80%–85% in the presence of CO_2_ and H_2_O as oxidants in place of O_2_. Mechanistic studies have shown that oxygen vacancies play an important role in the catalysis and regeneration process as the reaction progresses. Understanding the structures of these oxides is as important as their syntheses to οβταιν an understanding of the active sites of these materials and to correlate them with respective performances.

## 6 TMOs-based ceramics in high-temperature applications

Materials that have the capability to withstand high temperatures are in high demand in the aerospace industry. These materials are used in various applications such as the hot section of the gas turbine engine, brake pads, and as heat shields in space vehicles (Bakan et al., 2020; Binner et al., 2020; Borawski, 2020). Apart from being able to operate at high temperatures, these materials should also be resistant to extreme mechanical and oxidative stresses. A single material that adheres to all these criteria has not been invented yet. Instead, ceramic materials which have high temperature resistance are formed into composite materials known as Ceramic Matrix Composites (CMCs) to overcome the high mechanical and temperature stresses (Sato et al., 1999). A protective coating is added on top of the surface to protect these composites and nickel based superalloy structural parts from oxidation. This protective layer is known as Environmental Barrier Coatings (EBCs) for CMCs or Thermal Barrier Coatings (TBCs) for Nickel based superalloy structural parts (Lee, 2000; Vaßen et al., 2010). TMOs are promising candidates for making these protective coatings due to their low thermal conductivity, high thermal stability, and high corrosion resistance (Fang et al., 2023).

### 6.1 Ceramic Matrix Composites

**FIGURE 5:**
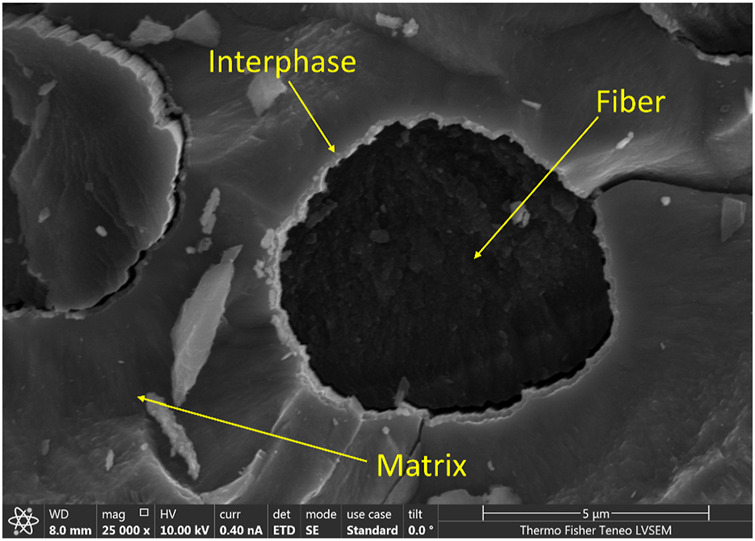
Cross sectional SEM image of carbon fiber with SiC coating of CMC.

CMCs consist of a three-part system namely Matrix, Interface, and Reinforcing Fiber. Bulk ceramic material is considered as the matrix. The matrix could be nonoxide ceramics like silicon carbide or oxide ceramics like aluminum oxide. Monolithic refractory ceramics which are used as the matrix are usually brittle. Reinforcing fibers are incorporated into the bulk ceramic material to increase the mechanical strength of the ceramic. Commonly used fibers are carbon fibers or silicon carbide fibers (Gottlieb et al., 2016). These fibers increase the strength of the final composite by carrying the mechanical load applied upon the composite. Figure 5 shows a cross sectional SEM image of a carbon fiber/SiC CMC which, is synthesized using chemical vapor deposition methods. It is imperative to protect these fibers from oxidation at high temperatures to avoid ultimate composite failure due to oxidation or crack propagation through fibers (Li, 2020). Prior to densifying the bulk matrix, fibers are coated with a thin layer of ceramic to overcome this scenario, and this thin layer is known as the interphase layer. Such interphases act as barriers to oxidation and stop failure of fibers by crack deflection or by facilitating fiber debonding (Kerans et al., 2002).

Oxidation under high temperatures is a major drawback in CMCs. Most nonoxide ceramics decompose into their respective oxides under high thermal stress leading to investigations of making CMCs using metal oxides (Coons et al., 2013). Poges et al. studied the effect of a transition metal zinc oxide as the interface coating in oxide/oxide CMCs by comparing the flexural strength of a CMC system containing Nextel 610 reinforcing fibers and an alpha alumina matrix with a ZnO interphase coating to the same CMC system without a ZnO interphase layer. From their data, adding the ZnO interphase layer increased the flexural strength by 30% for the CMC by introducing toughening mechanisms such as interphase debonding and crack deflection (Poges et al., 2017).

### 6.2 TMO ceramics as thermal/environmental barrier coatings

**FIGURE 6:**
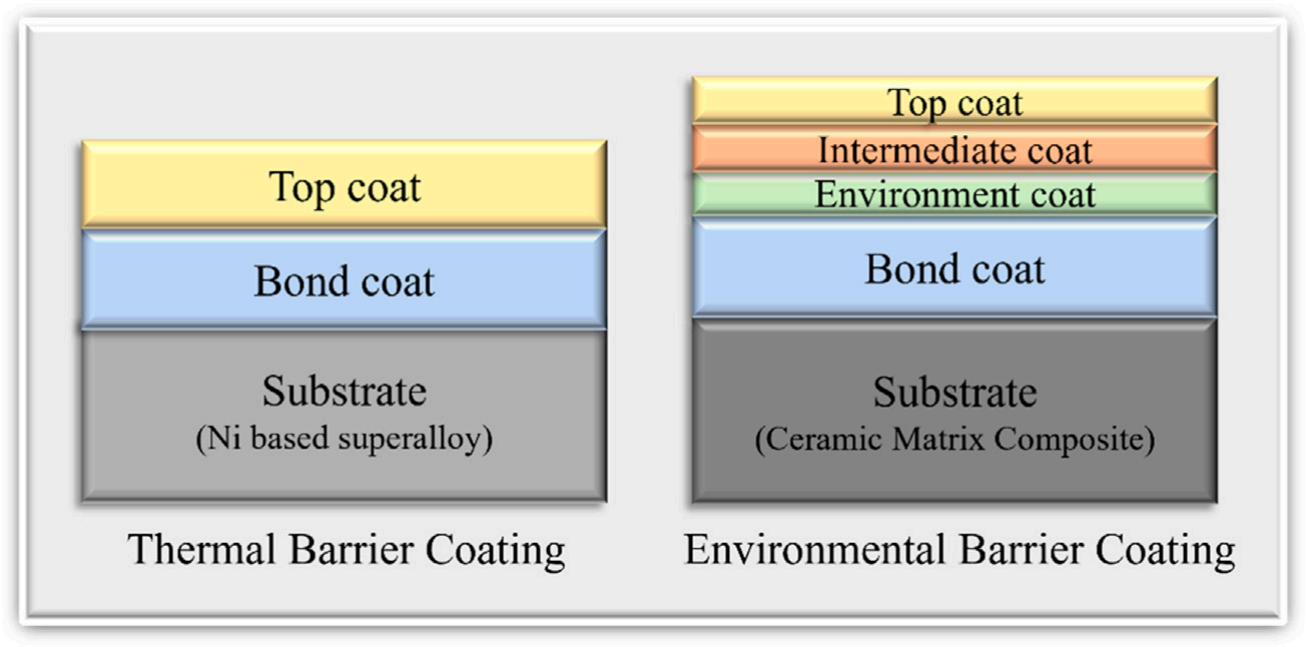
Schematic diagram of TBCs and EBCs (Thickness of the layers are not depicted in correct proportions).

In the aerospace industry thermal barrier coatings are applied on top of Nickel based superalloys for protection from high thermal stresses. TBCs consist of two layers, namely, the bond coat and the top coat as shown in Figure 6 (Mehta et al., 2022). Yttria stabilized Zirconia (YSZ) is the most used transition metal oxide ceramic in the aerospace industry which provides thermal resistivity due to its low thermal conductivity.

The behavior of YSZ under the cycling of thermal and oxidative stresses has been widely studied over the past couple of decades (Weng et al., 2020). With the emergence of more powerful next-generation gas turbine engines, the operating temperature of the hot section has risen considerably over the years. There have been various recent attempts to develop novel transition metal oxide systems that can outperform current YSZ systems.

Sang et al. developed a novel high entropy ceramic system (Sm_0.2_Lu_0.2_Dy_0.2_Yb_0.2_Y_0.2_)_3_TaO_7_ using a sol gel and sintering hybrid method with high crystallinity and increased phase stability. This transition metal oxide system has the single fluorite crystal system, and exhibits lower thermal conductivity than the YSZ system. The low thermal conductivity is due to the complexity of the elemental composition, oxygen vacancies, and lattice distortion which ultimately resulted in higher phonon scattering (Sang et al., 2023).

A recent study by Dang et al. suggested a novel candidate for the TBC top coat. They produced yttria doped hafnia (Y_4_Hf_3_O_12_) using an atmospheric plasma spray method. This material showed promising results for phase stability after 200 h at 1,300°C and thermal stability after 459 cycles. Phonon thermal conductivity measurements portrayed lower thermal conductivity than YSZ and authors attributed this lower thermal conductivity to the high concentration of Y cations in the fluorite crystal system. Even though this material has promising results, the low Coefficient of Thermal Expansion (CTE) is a downside. Y_4_Hf_3_O_12_ was suggested as coating with a YSZ buffer coat to overcome that problem (Dang et al., 2023).

Environmental barrier coatings are more complex when compared to thermal barrier coatings. Typically, third generation EBCs have four layers on top of the CMC substrate namely Topcoat, Intermediate Coat, Environment Coat, and Bond Coat. Oxide ceramics like mullite and BSAS (1-xBaO.xSrO.Al_2_O_3_.2SiO_2_,0 < x < 1) are widely used in top coats (Chen et al., 2023). In recent years there have been new developments of a new type of ceramic coating which involves transition metal elements. These are called rare earth monosilicates (RE_2_SiO_5_) or rare earth disilicates (RESi_2_O_7_) (Tejero-Martin et al., 2021).

Among the available rare earth silicates, Ytterbium disilicates (Yb_2_Si_2_O_7_) have caught the attention of the researchers due to their close CTE match with SiC substrates and the relatively high oxidation corrosion in Ytterbium monosilicates (YbSiO_5_). Wang et al. hypothesized that combining a mixture of these two Ytterbium silicates would be more successful as an EBC topcoat and studied the protection effects of the Atmospheric Plasma Spray deposited mix coating on SiC_f_/SiC composites. After exposing the system to a mixture of water vapor and oxygen at 1,300°C for 200 h the protected CMC systems retained their original strength by 90% when compared to the non-protected ones only retaining 10%–15% (Wang Y. et al., 2022).

Transition metal oxides are mostly used as protective coatings in the aerospace industry. For example, fabricating CMCs involves depositing a thin film coating on the reinforcing fibers, or making a TBC/EBC involves depositing a multilayer coating on substrates leading to the utilization of thin film deposition techniques. The most common thin film fabrication technique is chemical vapor deposition (CVD). In CVD the substrate is heated to the desired deposition temperature and then the precursors are flown over the substrate. When the precursors meet the heated substrate, they react and form a thin film (Morosanu, 1990). CVD is a very useful technique, but is also a highly inefficient technique. Most of the reactants will not react and form a thin film. The other problem is finding the proper precursor. Reactants must be either in gaseous form or should be able to be converted into gaseous forms by evaporation or sublimation. This limits the number of compounds that can be formed by CVD.

Various thin film coating techniques like electron beam physical vapor deposition, plasma spray, and magnetron sputtering are used to make TBC/EBCs. Among these methods, Atmospheric Plasma Spray (APS) is commonly used to make these complex multilayer protective coatings due to low cost, flexibility of the process, and higher deposition rates. In this method precursors are heated using plasmas and then mixed with carrier gas. Then the mixture is bombarded onto the pretreated substrate (Thakare et al., 2021). Due to the nature of the process, the coating is formed in a lamellar structure which induces the formation of amorphous phases and undesirable microstructures such as porosity. Post process annealing is required to overcome these challenges. This will allow the amorphous phases to crystallize and heal the microcracks and pores present in the coating (Arhami et al., 2023).

## 7 Challenges from theory in modeling TMOs

**FIGURE 7:**
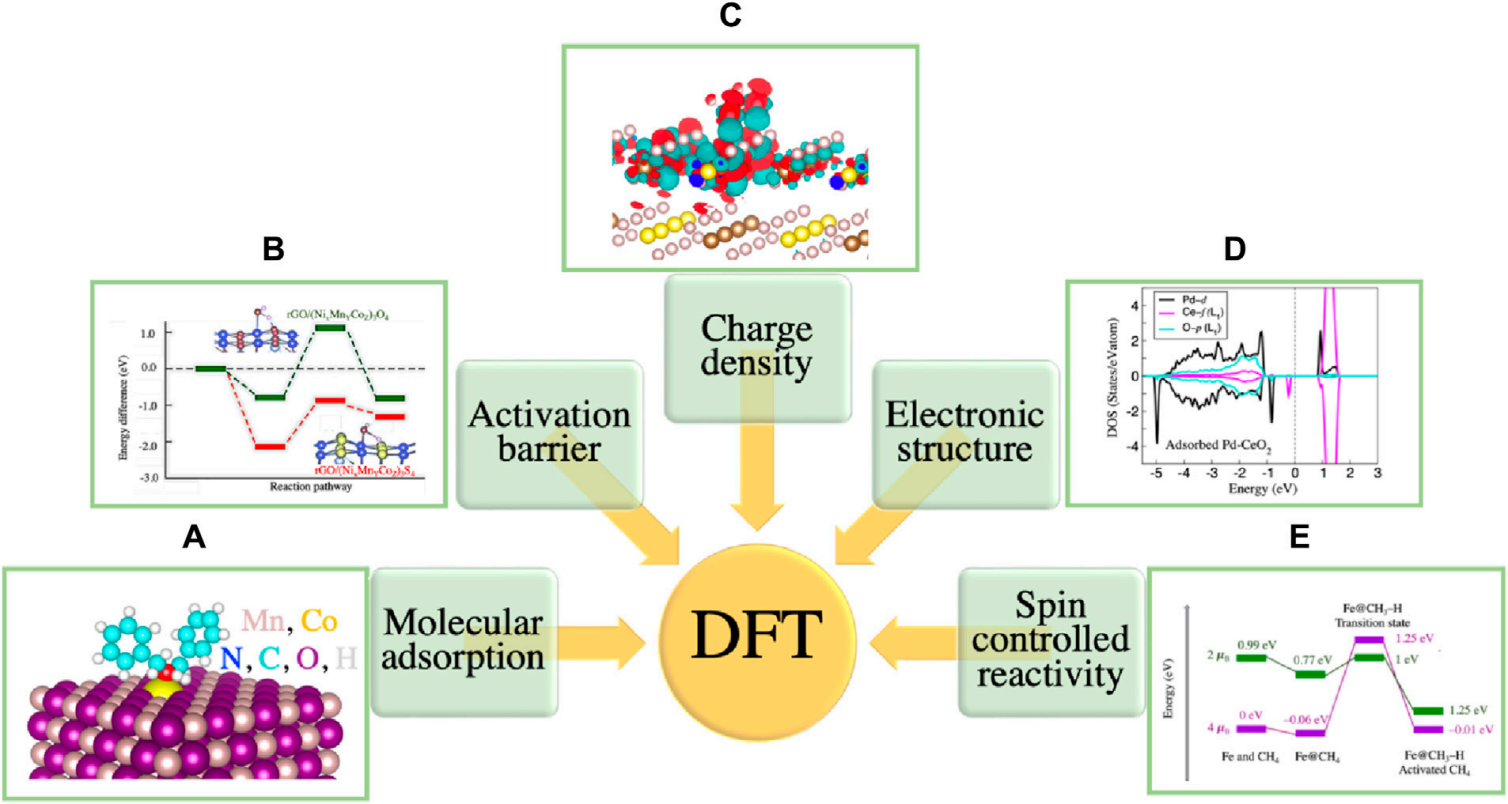
**(A)** Stable adsorption configuration of benzylamine molecule on meso-Co-MnO_x_(100) surface (Dutta et al., 2018). **(B)** Reaction pathways for water (O–H) bond breaking on spinel sulfide (red line) and spinel oxide (green line) surfaces with the corresponding transition state geometries (Miao et al., 2017a). **(C)** Charge density difference for adsorbed water on FeS_2_ (100) surface (Miao et al., 2017b). Red and turquoise balloons denote the charge accumulation and charge depletion regions, respectively. **(D)** The electronic density of states of Pd-CeO_2_(111) after H_2_ adsorption (Sahoo et al., 2021). **(E)** Methane cracking reaction demonstrating the multi-state reactivity with Fe catalyst Sahoo et al. (2016).

The bonding and interaction of reactants with the surface and the influence of surfaces on the bonding and interactions between reactants are the key factors that significantly contribute to catalytic processes. The TMO surface imposes several complexities for both theoretical modeling and experimental characterization. Some of the important descriptors that must be taken into account for surface characterization and surface reactions are: the stoichiometric ratios of cations and anions, the covalent and ionic bonding, the charges of adsorbed species, the surface acidity and basicity, the cation and anion vacancies, oxygen mobility, and the surface reconstruction due to the presence of dangling bond. These complexities make TMOs one of the most complicated systems for experimental and theoretical investigations. While recent experimental techniques can synthesize and characterize TMO surfaces, understanding the atomic level interactions in the TMOs is extremely challenging from an experimental viewpoint. For instance, obtaining information about processes related to the diffusion and mobility of impurities and oxygen, the locations and the amounts of oxygen vacancies, the charge transfer mechanism, the exact electronic structures, and the surface reconstruction processes are extremely challenging. Furthermore, contamination of the surface with light-weight elements (below the atomic number of Na) that can easily interfere with surface reactions and influence efficiency, is hard to track in laboratories without the application of numerous special techniques. Electronic structure methods have been proven to be highly effective to address these issues. The accuracy and efficiency of these methods has led to a paradigm shift in coupling experiments and theoretical research together with the aim of tangible understanding, predicting, and designing properties of materials. Several quantum chemical methodologies, such as wavefunction-based Hartree–Fock (HF), and post-Hartree–Fock approaches ranging from Møller–Plesset (MP) perturbation theory (Møller and Plesset, 1934) to coupled cluster (CC), configuration interaction (CI) (David Sherrill et al., 1999), and many body Green’s function methods have been applied to study catalysis (Mandaliya and Gudi, 2023). The focus of our modeling has been the total charge-density based density functional theory (DFT) which helps to track chemical reactions with a larger size of model that is representative of the actual experiments (Kohn and Sham, 1965; Jones, 2015). The quantities related to chemical reactivity and catalysis such as the molecular adsorption sites on surfaces, the binding energies between adsorbates and surfaces, the reaction pathways and activation barriers, the charge distribution and charge tr100ansfer are also reliably predicted using DFT methods (Sahoo et al., 2018a; Sahoo et al., 2021; Bamonte et al., 2022). Figures 7A–E demonstrates examples of systems with such properties. More details are explained in the caption.

Although DFT is limited to zero temperature and pressure, it has a major role for investigating the thermal stability and composition of transition metal surfaces under realistic conditions. One such approach is the *ab initio* thermodynamics method where the total energies obtained from DFT are used as input for determining the thermodynamic phase stability as well as the surface structure and composition (Reuter et al., 2005; Stampfl, 2005; Sahoo et al., 2018b).

For example, when transition metal surfaces are exposed to high oxygen pressures, thin oxide-like structures are reported to form with little similarity to the bulk oxides (Stampfl, 2005). Among these, oxygen interaction with Ag(111), Pd(111), Ru(0 001) and Rh(111) surfaces are important. For O/Ag(111) and O/Pd(111), the free energy phase-diagrams as a function of (*T*, *p*) show high stability for thin surface-oxide-like configurations whereas, the O/Rh(111) and O/Ru(0001) are metastable with respect to bulk oxide formation. In many of these cases, formation of surface-oxides leads to significant geometrical changes with little similarity to the corresponding bulk oxide (Stampfl, 2005). Using a similar approach, the interaction of oxygen, nitrogen and hydrogen with Ti(0001) surfaces under varying gas partial pressure and temperature is studied, where the Ti(0001) surface was reported to have the high affinity for oxygen with a strong surface passivation tendency compared to nitrogen and hydrogen (Sahoo et al., 2018b). Apart from *ab initio* thermodynamics, there are other methods such as the Kinetic Monte Carlo (KMC) and microkinetic modeling where the DFT total energies could be used as inputs for investigating the reaction kinetics under realistic conditions (Kattel et al., 2017b).

In TM catalysis, spin controlled reactivity is an important characteristic. DFT offers a versatile framework for investigating the multistate reactivity via providing detailed description of electronic transitions between different molecular states and insights into the electronic structure as well as energetics of complex chemical processes. For instance, during a reaction the reactants in a given spin state could convert into products with the same spin state, but the intermediates could have a different spin state. One such example is shown in Figure 7E where the interaction of methane with Fe catalyst has demonstrated a multi-state reactivity for the methane cracking reaction (Sahoo et al., 2016). The minimum energy pathway for methane activation is characterized by two spin states, a high spin state of 4 μ_B_, and a low spin state of 2 μ_B_. The high spin state is more stable for the reactant complex by 0.99 eV, as the reaction progresses, a spin-crossover takes place at the transition state with methane C-H bond distance (1.58 Å). The low spin state becomes favorable followed by a high spin state as energetically stable for the product (the cracked methane with C-H bond distance 2.93 Å). With the evolution of computational methodologies, DFT is expected to play a key role in unraveling the intricate mechanisms of multistate reactions, guiding experimental efforts, and contributing to the design of novel materials and catalysts for applications in diverse fields of chemistry and materials science.

Given certain limitations of DFT pertaining to strong electron correlation effects that describe the properties of TMOs, one needs certain technical improvements in DFT to accurately capture the properties. The most used approach is the Hubbard U treatment to DFT Hamiltonian, called the DFT+*U*. The parameter *U* is either calculated theoretically or determined from experiments by matching the electronic density of states. The DFT + *U* has been found to yield accurate and robust results (Adeagbo et al., 2014; Nayak et al., 2015; Miao et al., 2017a; Miao et al., 2017b).

Catalysis studies have been done through a hybrid-functional approach to the DFT, where while the correlation interactions are kept at the generalized-gradient approximation level, the exchange interaction is supplemented by screened Hatree–Fock exchange constructed from the Kohn–Sham orbitals. Such an approach has shown to improve the band gap description of bulk semiconductors and strong-correlation of the TMO almost simultaneously. The hybrid functional scheme has been applied to study surface reactions (Getsoian and Bell, 2013). However, owing to their intense computational resource requirements, this method is not the most applied method in the community. Furthermore, questions about how to treat the reference energies in the formation energy calculations (Nayak et al., 2015; Nayak et al., 2018; Reid et al., 2020), and reliable modeling of electronic structures for more sophisticated quantum properties, suggest that the hybrid functional approach may be redundant for high-Z TMOs. As shown in the literature, spin orbit coupling is more Important to describe these delicate properties.

The optimal choice of the functional in DFT ultimately depends on the target properties and requires validation with experiments. However, the analogy is not universal across all materials. Identifying and understanding relationships between the electronic and atomic structure of surfaces and their catalytic activity is an essential step towards the rational design of heterogeneous catalysts. One successful example is the *d*-band model for transition metals, which has provided rational trends in surface reactivity with compositional changes, lattice constants, and facets. The *d*-band model has also provided a physical basis for the widely used descriptor-based analyses and scaling relationships between adsorbate binding energies and transition state energies (Dahl et al., 2001; Xin et al., 2014).

**FIGURE 8:**
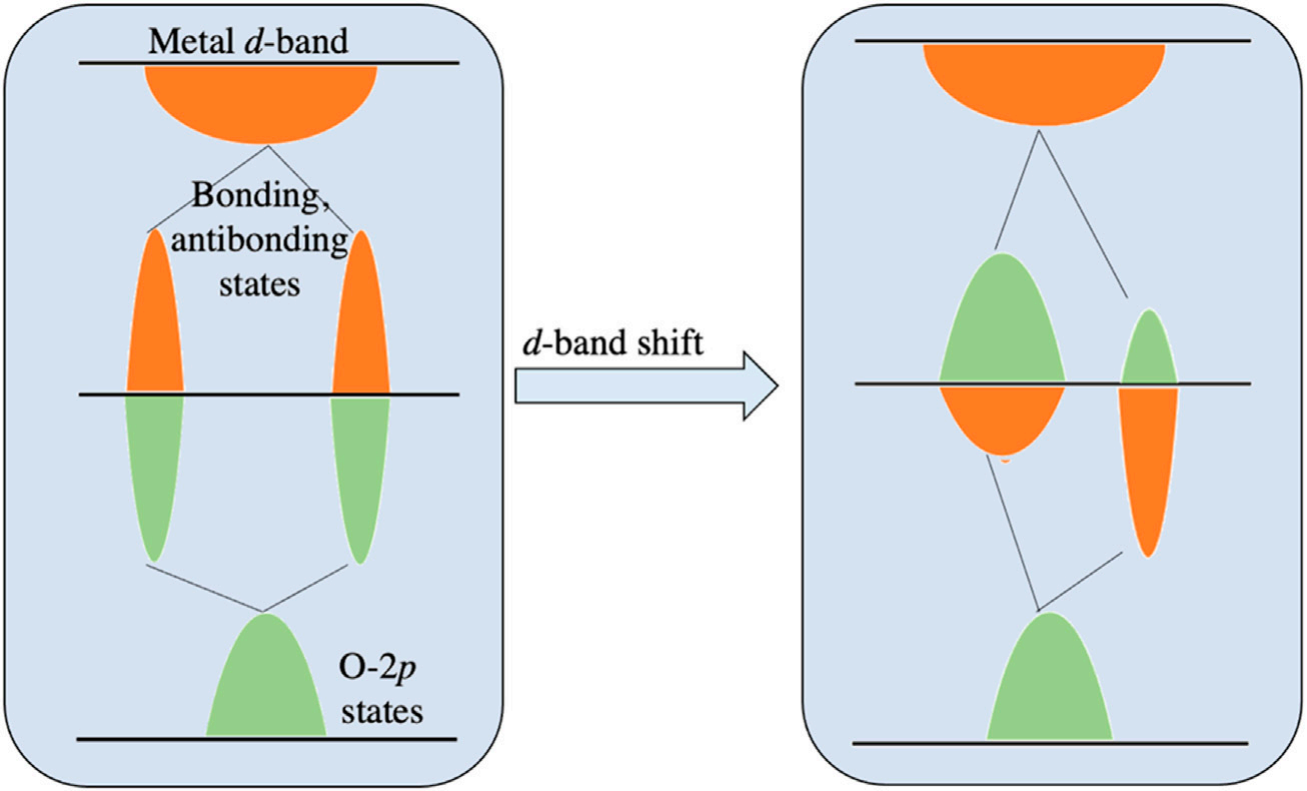
The energy diagram demonstrating the change in metal *d*-band in the 2*p*-states of adsorbed oxygen at a transition metal surface. As shown in the left panel, prior to the mixing with metal *d*-band, the O 2*p*-states hybridize with the metal *sp*-band, resulting in the O 2*p*-state. These states interact with the metal *d*-band as shown in the right panel, to form bonding and antibonding states, which are projected onto the O 2*p*-states or metal *d*-states as indicated by the green peaks and orange peaks, respectively.

Recently, for TMOs, a direct relationship is established between electronic structure and catalytic properties, where the correlations between adsorbate binding energies and the metal *d*-states or oxygen 2*p*-states have been made for rutile, perovskite, and rock-salt TMOs (Dickens et al., 2019). The oxygen electrochemical rates of perovskite catalysts, have been shown to correlate with the electron occupancies and other electronic structural properties such as the oxygen 2*p*-band center and charge transfer energy obtained from DFT and X-ray spectroscopy measurements. A description in this regard is given in Figure 8.

## 8 Conclusion

The review provides an overview of designing TMOs and their functional applications using state-of-the-art synthetic and characterization techniques, with specific emphasis on mesoporous oxides. The primary focus on the applications side includes utilizing TMOs as effective materials for clean energy and sustainability, especially involving their usage in photocatalysis, electrocatalysis. Another usage includes their application in high-temperature thermal barrier coatings that is important in aerospace industry. Despite numerous studies, the characterization and investigation of TMOs, both from experimental and theoretical perspectives, have not yet reached an optimum level. There are still many important issues and open questions that require attention for a complete understanding of TMOs. A key research focus pertains to the development of theoretical methods capable of providing reliable energies and an accurate description of the electronic structure of TMOs. In this regard, DFT, has become an important tool for supporting the interpretation of experimental data such as providing structural insights, analyzing spectroscopic data, and testing mechanistic hypotheses through the microscopic insights into the relationships between atomic and electronic structures. The review addresses the major pros and cons of DFT as well as those issues that need to be overcome in modeling TMOs to achieve the desired functionalities and optimization of TMO-based catalysts.
